# A multinational survey of potential participant perspectives on ocular gene therapy

**DOI:** 10.1038/s41434-024-00450-4

**Published:** 2024-04-02

**Authors:** Alexis Ceecee Britten-Jones, Myra B. McGuinness, Fred K. Chen, John R. Grigg, Heather G. Mack, Lauren N. Ayton

**Affiliations:** 1 Department of Optometry and Vision Sciences, Faculty of Medicine, Dentistry and Health Sciences, University of Melbourne, Parkville, VIC Australia; 2 Ophthalmology, Department of Surgery, University of Melbourne, Melbourne, VIC Australia; 3 Centre for Eye Research Australia, Royal Victorian Eye and Ear Hospital, Melbourne, VIC Australia; 4 Centre for Epidemiology and Biostatistics, Melbourne School of Population and Global Health, University of Melbourne, Melbourne, VIC Australia; 5 Centre for Ophthalmology and Visual Sciences (incorporating Lions Eye Institute), The University of Western Australia, Perth, WA Australia; 6 Royal Perth Hospital and Perth Children’s Hospital, Perth, WA Australia; 7 Save Sight Institute, The University of Sydney, Sydney, NSW Australia; 8 Eye Genetics Research Unit, Sydney Children’s Hospitals Network, Save Sight Institute, Children’s Medical Research Institute, University of Sydney, Sydney, NSW Australia

**Keywords:** Diseases, Health sciences, Genetics

## Abstract

Amidst rapid advancements in ocular gene therapy, understanding patient perspectives is crucial for shaping future treatment choices and research directions. This international cross-sectional survey evaluated knowledge, attitudes, and perceptions of ocular genetic therapies among potential recipients with inherited retinal diseases (IRDs). Survey instruments included the Attitudes to Gene Therapy-Eye (AGT-Eye), EQ-5D-5L, National Eye Institute Visual Functioning Questionnaire (NEI-VFQ-25), and Patient Attitudes to Clinical Trials (PACT-22) instruments. This study included 496 participant responses (89% adults with IRDs; 11% parents/guardians/carers) from 35 countries, with most from the United States of America (USA; 69%) and the United Kingdom (11%). Most participants (90%) indicated they would likely accept gene therapy if it was available, despite only 45% agreeing that they had good knowledge of gene therapy. The main sources of information were research registries (60% of participants) and the internet (61%). Compared to data from our recently published Australian national survey of people with IRDs (*n* = 694), USA respondents had higher knowledge of gene therapy outcomes, and Australian respondents indicated a higher perceived value of gene therapy treatments. Addressing knowledge gaps regarding outcomes and financial implications will be central to ensuring informed consent, promoting shared decision-making, and the eventual clinical adoption of genetic therapies.

## Introduction

Voretigene neparvovec-rzyl (Luxturna®) for treatment of biallelic *RPE65*-related inherited retinal diseases (IRDs) was the first Food and Drug Administration (FDA)-approved ocular gene therapy treatment [1], with subsequent regulatory approval in the UK, Europe, Australia, and other global regions. Following its approval, gene therapy treatments are being developed for other rare monogenic IRDs [2], as well as more common ocular diseases, including age-related macular degeneration and glaucoma [3, 4]. In addition to gene replacement therapy, other emerging genetic treatments, including RNA therapies, CRISPR-repaired stem cell therapies, and optogenetic treatments, are also being developed targeting different stages of disease and vision loss [5].

A level of understanding of these emerging biotechnologies is vital to ensure participants’ ability to make informed decisions about receiving these treatments and to facilitate translation of these therapies into clinical applications. Understanding community knowledge of ocular gene therapies is also important for developing evidence-based communication strategies and can assist clinicians in facilitating shared decision making [6]. In a recent Australian national survey of IRD participants, we found a high level of interest towards ocular gene therapies among 681 respondents, with 92% indicating that they would likely accept gene therapy if it was available to them or their family member, despite only 28% agreeing that they had good knowledge of gene therapy. However, it is not clear how knowledge and perceptions of genetic therapies might differ among IRD communities in other global regions.

From two reviews that systematically evaluated perspectives on non-ophthalmic cell and gene therapies, members of the public generally expressed acceptance, with some geographical variation [7, 8]. In stem-cell research, a multinational survey found that in the USA moral acceptability was more influential as a driver of support, whereas in Europe the perceived benefit to society carried more weight, and in Canada both were rated equally important [9]. However, these studies did not specifically capture patient views on reimbursement.

The aims of this multinational cross-sectional study were to evaluate the knowledge, attitudes, and perceptions of ocular gene therapies among IRD patients and/or carers, and to assess geographical variations by comparison with recently published data from an Australian national IRD survey [10].

## Methods

Ethical approval was obtained from the University of Melbourne Human Research Ethics Committee (#2023-25634-38542-4). This study was undertaken in accordance with the principles of the Declaration of Helsinki and with the Australian National Statement on Ethical Conduct in Human Research. All participants consented to participating prior to undertaking the survey.

An online cross-sectional structured survey was administered following the same protocol previously described for an Australian cohort [10, 11]. Eligible participants were aged 18 years and above and either had a self-reported IRD, were the parent/guardian of a child or dependent aged under 18 years with an IRD, or were a caregiver for an adult with an IRD. Included IRDs and excluded conditions are shown in Supplementary Table 1. Carriers of IRDs without an ocular phenotype and people with complex retinal conditions (such as age-related macular degeneration) in the absence of a clinically diagnosed IRD were ineligible to participate. As the party responsible for making treatment decisions, parents/caregivers were asked to give their own response, rather than the answer they believed their dependent would give. Participants from any country eligible to participate. The survey was administered in English.

Demographic information was collected, and participants were asked about previous participation in medical research, their likelihood of taking up gene therapy treatment if it was available to them or their dependents, and their perceived barriers to receiving gene therapy. Participants then responded to the following instruments in sequence (i) Attitudes to Gene Therapy-Eye (AGT-Eye) survey [12]; (ii) EuroQoL EQ-5D-5L questionnaire (Australian English version) [13], to assess overall quality of life; (iii) National Eye Institute Visual Functioning Questionnaire (NEI-VFQ-25; Australian English version) [14], to evaluate vision-targeted health status; and iv) PACT-22 Clinical Trial Attitudes Scale [15].

The 22-item AGT-Eye survey [10–12], developed by the study authors, assesses participants knowledge and expectations of potential recipients of ocular gene therapy for IRDs, with responses rated on a five-point Likert scale from 1 (Strongly disagree) to 5 (Strongly agree). Psychometric properties of AGT-Eye were previously investigated using item response theory methodology, resulting in four subscales: Sources of information, Knowledge of methods, Awareness of outcomes, and Perceived value of treatment [12]. The AGT-Eye questionnaire was developed in consultation with an IRD participant committee, as previously reported [11]. Following the Australian survey, several AGT-Eye items in the current version were updated to improve interpretability, taking into account feedback from IRD participants [11]. Changes from original instrument wording as shown in Supplementary Table 2.

AGT-Eye subscale scores were compared with each of NEI-VFQ-25, EQ-5D-5L, and PACT-22 scores to evaluate the association between participants’ perception of ocular gene therapy, and both health- and vision-related quality of life and attitudes towards clinical trials. The EQ-5D-5L and NEI-VFQ-25 instruments were scored according to published methods [12–14]. For PACT-22, each question was scored between 1 (Strongly disagree) and 5 (Strongly agree) and subscale scores were calculated as the mean of item responses and standardized to a scale from 0 (high level of disagreement) to 100 (high level of agreement). Items relating to negative expectations (13–18) were reverse coded so that higher scores corresponded with more positive attitudes. EQ-5D-5L utility scores were derived from the United States value set and scoring algorithm, and visual analogue scale (VAS) scores were reported out of 100 [16]. For assessment of quality-of life instruments (NEI-VFQ-25 and EQ-5D-5L), parents/caregivers/carers were asked to complete the survey according to their own views and not on behalf of their dependent. Copyright owners of the other validated instrument provided permission for their use.

De-identified data were collected directly on Research Electronic Data Capture (REDCap) hosted at Centre for Eye Research Australia. REDCap is a secure web application for building and managing online surveys and databases [17].

### Recruitment

The REDCap open survey link was distributed to potential respondents through several international patient support and advocacy groups (Foundation Fighting Blindness, Retina International, Choroideremia Foundation, Stargardt’s Connected, and Eyes on the Future). These groups are based in the USA, UK, or Ireland, but all have international membership. The survey was distributed to members of the organisations via emails or through electronic newsletters and promoted on the organisations’ social media pages (Facebook, LinkedIn, and X, formerly known as Twitter).

### Statistical analysis

The primary analysis set included all eligible participants who completed the demographic and AGT-Eye sections of the survey, with data from Australian respondents removed to enable an unbiased comparison of global respondents and the data previously collected from an Australian national IRD survey [10]. Non-random convenience sampling was used. A sample size was not chosen to detect a pre-specified effect size.

Participant characteristics are presented according to respondent status (adult with IRDs vs parent/guardians/caregivers), as mean and standard deviation (SD) for variables with an approximately normal distribution, median and interquartile range (IQR) for variables with a skewed distribution, and frequency and percent for categorical variables.

AGT-Eye subscale scores were compared between participant characteristics; patient vs carer, age (18–39, 40–59, or 60+ years), gender (male, female, or non-binary), type of IRD (central or widespread), highest level of education (primary school, secondary school, trade certificate, bachelor degree, or postgraduate degree), self-reported vision (excellent, good, fair, poor, very poor, completely blind), and likelihood of taking up gene therapy (likely, neutral, or not likely) using one-way analysis of variance tests. Subscale scores were not presented for characteristic categories with <5 participants due to disclosure risk. Spearman’s correlation coefficient was calculated for the relationship between AGT-Eye and each of PACT-22 subscale scores, NEI-VFQ-25 subdomain scores, and EQ-5D-5L and EQ-VAS scores. The comparison between AGT-Eye and PACT-22 scores included participants with non-missing scores for all instruments, and between AGT-eye and the quality-of-life instruments included only adults with an IRD who completed both questionnaires.

International responses were summarised by country of residence (USA vs Other) and compared with a previously published complete case set for an Australian national survey of people with IRD (*n* = 681) [10]. As the USA constituted the largest international survey cohort, post-hoc comparison of AGT-Eye responses were only undertaken between respondents from the USA and Australia. Response frequencies were compared between countries using Pearson’s Chi-squared test. Subscale scores were compared using two-sample *t* test (two groups) or one-way analysis of variance (three groups). For comparison of Australia versus USA data, multiplicity adjustments using the Bonferroni correction were calculated using a family wise type I error probability of 5%.

Statistical analyses were conducted using Stata/BE v18.0 (StataCorp, College Station, Tx) and R for statistical consulting (v4.2.2; R Core Team 2021).

## Results

Between 13 April and 25 July 2023, 699 participants provided consent. Of these, 502 eligible respondents completed the demographics and AGT-Eye questionnaire, and 477 respondents completed all survey instruments (Supplementary Fig. 1). After excluding participants residing in Australia (*n* = 6), data from 496 participants were included in the primary analysis set (Participant characteristics shown in Table 1 and Supplementary Table 3).

**Table 1:** Participant characteristics of the multinational survey cohort (*n* = 496).

	Participant type		
	Adult with IRD	Parent/guardian/carer	Total
	*n* = 439 (88.5%)	*n* = 57 (11.5%)	*n* = 496 (100.0%)
Age (years)	55.6 (14.4)	45.3 (11.0)	54.5 (14.5)
Gender			
Male	219 (49.9%)	18 (31.6%)	237 (47.8%)
Female	216 (49.2%)	39 (68.4%)	255 (51.4%)
Non-binary	2 (0.5%)	0 (0.0%)	2 (0.4%)
I prefer not to say	2 (0.5%)	0 (0.0%)	2 (0.4%)
Age of first symptoms (years)	24 [12, 40]	5 [1, 12]	20 [10, 38]
Primary language			
English	393 (89.5%)	37 (64.9%)	430 (86.7%)
Other than English	46 (10.5%)	20 (35.1%)	66 (13.3%)
Country of residence			
USA	316 (72.0%)	32 (56.1%)	348 (70.2%)
UK	50 (11.4%)	5 (8.8%)	55 (11.1%)
Other	73 (16.6%)	20 (35.1%)	93 (18.8%)
Ethnicity			
White	379 (86.3%)	50 (87.7%)	429 (86.5%)
Hispanic or Latino	19 (4.3%)	4 (7.0%)	23 (4.6%)
Asian	22 (5.0%)	2 (3.5%)	24 (4.8%)
Black or African American	10 (2.3%)	1 (1.8%)	11 (2.2%)
American Indian or Alaskan Native	4 (0.9%)	0 (0.0%)	4 (0.8%)
I don’t know/Prefer not to say	5 (1.1%)	0 (0.0%)	5 (1.0%)
IRD diagnosis			
Retinitis Pigmentosa	272 (62.0%)	20 (35.1%)	292 (58.9%)
Choroideremia	14 (3.2%)	2 (3.5%)	16 (3.2%)
Cone-rod Dystrophy	21 (4.8%)	4 (7.0%)	25 (5.0%)
Stargardt Disease	57 (13.0%)	10 (17.5%)	67 (13.5%)
Leber Congenital Amaurosis	8 (1.8%)	15 (26.3%)	23 (4.6%)
Macular Dystrophy	28 (6.4%)	2 (3.5%)	30 (6.0%)
Other central condition	7 (1.6%)	3 (5.3%)	10 (2.0%)
Other widespread condition	32 (7.3%)	1 (1.8%)	33 (6.7%)
Highest level of education completed			
Primary school (up to 12 years of age)	15 (3.4%)	3 (5.3%)	18 (3.6%)
Secondary school (until at least 15 years of age)	51 (11.6%)	9 (15.8%)	60 (12.1%)
Trade certificate	68 (15.5%)	3 (5.3%)	71 (14.3%)
Bachelor degree at a University	145 (33.0%)	23 (40.4%)	168 (33.9%)
Post-graduate degree at a University	144 (32.8%)	15 (26.3%)	159 (32.1%)
I prefer not to say	16 (3.6%)	4 (7.0%)	20 (4.0%)
Previously supplied DNA to an IRD database	310 (70.6%)	39 (68.4%)	349 (70.4%)
Previously participated in medical research	120 (27.3%)	13 (22.8%)	133 (26.8%)

Data are presented as Mean (Standard deviation) for age, Median [IQR] for age of first symptoms, and frequency (%) for all other variables. IRD=inherited retinal disease.

**Fig. 1:**
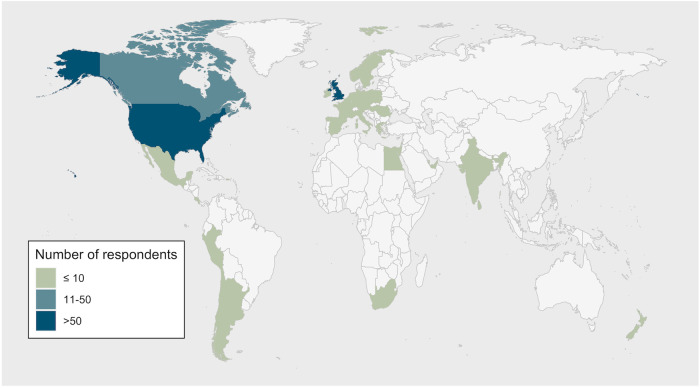
Country of residence of participants who participated in the multinational survey (*n* = 496). Respondents from Australia (*n* = 6) were excluded from the analysis.

Data included participants from 35 countries (Fig. 1). Most responses were from high income countries (96%), with respondents from the United States of America (USA; 69%) and United Kingdom (UK; 11%) constituting 80% of the study sample. English was the primary language reported by 87% of participants.

Most responses were from adults with IRDs (89%; *n* = 439; mean age 55.6 [14.4] years); 7% of respondents were parents/guardians/carers of people with IRDs <18 years of age (*n* = 36; mean age 44.8 (8.6) years), and 4% were parents/guardians/carers of people with IRDs >18 years of age (*n* = 36; mean age 46.2 (14.5) years).

**Fig. 2:**
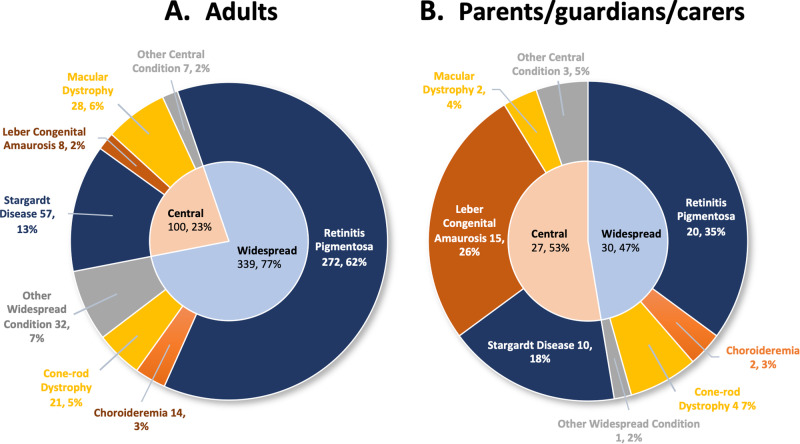
Self-reported diagnoses of survey respondents (*n* = 496). Responses shown from adults with IRDs (*n* = 439) or as reported of their dependent by parent/caregivers (*n* = 57).

Adult respondents predominantly reported having retinitis pigmentosa (62%), Stargardt disease (13%), or other macular dystrophies (6%); 2% of adult respondents had Leber Congenital Amaurosis (Fig. 2). The main presenting symptoms reported at disease onset were difficulty seeing at night/dusk (66%) and difficulty adjusting from light/dark (49%; Supplementary Table 3). In contrast, parents/guardians/carer responders were largely of dependents with retinitis pigmentosa (35%), Leber Congenital Amaurosis (26%), and Stargardt disease (18%).

**Fig. 3:**
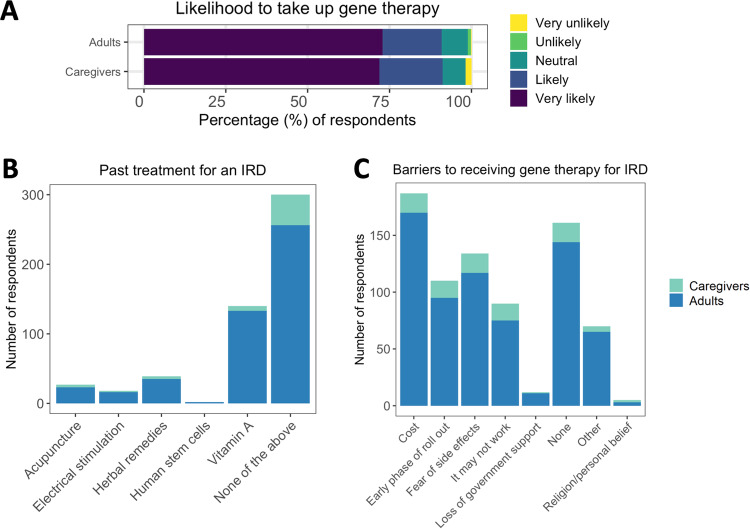
Participants’ attitudes towards gene therapy and medical research (*n* = 496). **A** Likelihood of taking up gene therapy if offered for their condition. **B** Previous treatment for inherited retinal disease. **C** Perceived barriers to receiving gene therapy for inherited retinal diseases. IRD inherited retinal disease.

Figure 3 shows participants’ attitudes towards gene therapy and medical research. Only 27% of participants have previously participated in medical research, but 70% have supplied DNA to an IRD database. Most respondents (61%) indicated that they had never previously received any treatments for their (or their dependent’s) IRD (Fig. 3B). Those who had mostly had vitamin A treatments (28% of all respondents), and less commonly herbal remedies (8%), acupuncture (5%), and electrical stimulation (4%). Two adult participants with IRDs (0.4% of all responses) had previously received stem cell treatment.

Over 90% participants said that they were very likely (73%) or likely (18%) to take up gene therapy if this was available to them (Fig. 3A). Only 6 participants (1.2%) said that they were unlikely or very unlikely to take up ocular gene therapy. Approximately one-third of respondents (32%) reported no barriers to getting gene therapy (Fig. 3C). The main barriers among both adults with IRDs and parents/guardians were cost of treatment (38% of all respondents), fear of side effects (27%), early phase roll-out of treatment (22%), and fear that treatment may not work (18%). Other barriers include lack of awareness about available treatments for their IRD or inadequate discussion of options (9%), causative gene has not been identified from genetic testing (1%), limited ability to travel for treatment access, fear of further vision loss, and apprehension about ineligibility for future treatments (each <1% of respondents).

### Attitudes to gene therapy (AGT-Eye) item scores

#### Sources of information

**Fig. 4:**
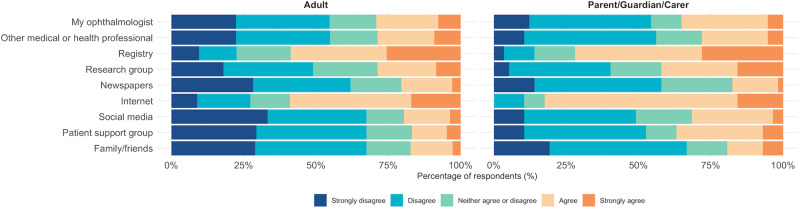
Reported sources of information about gene therapy. Sources reported from adults with IRD (*n* = 439), compared to parent/guardian/carers of a dependent with an IRD (*n* = 57).

Responses to all AGT-Eye items are shown in Supplementary Table 4. Figure 4 shows the sources where participants obtained information about gene therapy, which were primarily research registries (e.g., the Foundation Fighting Blindness registry; 60% participants) and internet (61% of all participants). Among all respondents, less than a third agreed that they received information about gene therapy from each of their ophthalmologist (30%), other medical or health professionals (28%), research groups (30%), or patient support groups (19%). Other sources of information, including social media (21%) and family/friends (17%), were less common.

Both parent/guardians (compared to affected adults) and participants who were more likely to take up gene therapy had higher sources of information subscale scores, indicating that they were more likely to have obtained information about gene therapy from different sources (Fig. 4). There were no differences between sources of information subscale score between age groups, gender, education levels, and self-reported vision status (Supplementary Table 5).

#### Knowledge of gene therapy methods

Despite most participants understanding the difference between an experimental treatment provided in a clinical trial and a treatment that has already been approved by the government (93%), less than half thought they had a good knowledge of gene therapy (45% agreed/strongly agreed).

**Fig. 5:**
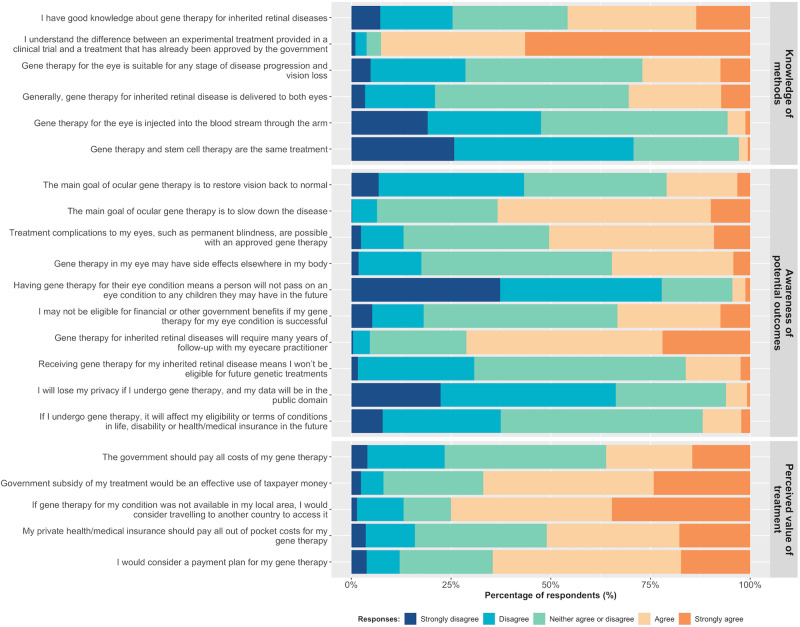
Participant responses to the attitudes to gene therapy for the eye questionnaire (*n* = 496). Response frequencies shown for the knowledge of methods, awareness of potential outcomes, and perceived value of treatment subdomains.

In the “knowledge of methods” subdomain (Fig. 5), 71% of all respondents knew that gene therapy and stem cell therapy are not the same treatment. However, only 47% knew that gene therapy for the eye is not injected into the blood stream through the arm, while a similar number of respondents (47%) neither agreed nor disagreed, indicating that they are uncertain of the details of the treatment delivery method. Less than a third of all respondents were aware that gene therapy for the eye may not be suitable for all stage of disease (29%), while 31% agreed/strongly agreed with the statement that generally, gene therapy for inherited retinal disease is delivered to both eyes.

Adult participants with IRDs (compared to parent/guardians) and participants with higher levels of education scored relatively higher in the “knowledge of methods” subscale. There was no difference in subscale scores between age groups, gender, central/widespread IRD, self-reported eyesight levels, or by participants’ likelihood of taking up gene therapy (Supplementary Table 5).

#### Awareness of potential gene therapy outcomes

Most participants agreed that the main goal of ocular gene therapy is to slow down the disease (63%). However, in the next question, over half (53%) also indicated that the main goal of ocular gene therapy is to restore vision back to normal (Fig. 5). Half (51%) agreed that treatment complications, such as permanent blindness, are possible with an approved gene therapy, and 35% knew that ocular gene therapy treatments can have side effects elsewhere in the body.

Most (77%) participants agreed that having gene therapy for their eye condition does not stop them passing on the gene to their children, and agreed that gene therapy for IRDs will require many years of follow-up with their eyecare practitioner (70%). However, approximately half of all respondents were not sure if: (i) receiving gene therapy for their IRD means they wouldn’t be eligible for future genetic treatments (52%); (ii) if they undergo gene therapy, it would affect their eligibility or terms of conditions in life, disability or health/medical insurance in the future (50%); and (iii) they might not be eligible for financial or other government benefits if gene therapy for their eye condition is successful (48%).

Younger participants and those who were more likely to accept gene therapy scored higher in the AGT-Eye’s “awareness of outcomes” subscale. There were no other differences in the “awareness” subscale scores between respondent type, gender, central/widespread IRD, self-reported eyesight levels (Supplementary Table 5).

#### Perceived value of treatment

Over 75% of participants would consider travelling to another country to access gene therapy for their condition, if it was not available in their local area. While two-thirds (67%) of respondents agreed that government subsidy of ocular gene therapy would be an effective use of taxpayer money, only one-third (36%) indicated that the government, and half (51%) that private health/medical insurance, should pay for all associated costs of ocular gene therapy treatment. While 65% of respondents would consider a payment plan for their gene therapy, 12% disagreed.

Perceived value of treatment was related to participants’ age, education levels, and likelihood of taking up gene therapy, as younger participants, those with higher levels of education, and those more likely to take up gene therapy had higher subscale scores. There were no differences in the perceived value of treatment scores between adults with IRD/parent guardians, gender, central/widespread IRD, self-reported eyesight levels (Supplementary Table 5).

### Relationship between AGT-Eye and other instrument scores

There was a weak to moderate correlation between each of the AGT-Eye subscale scores (Supplementary Table 6), with the strongest correlation between knowledge of methods and each of information sources (ρ = 0.50 [95% CI: 0.43–0.56]), awareness of outcomes (ρ = 0.29 [95% CI: 0.20–0.37]), and perceived value of treatment (ρ = 0.19 [95% CI: 0.10–0.27]).

Supplementary Tables 7–10 show the distribution of NEI-VFQ-25, PACT-22, and EQ-5D-5L scores and their correlations with AGT-Eye subscale scores. The median NEI-VFQ-25 composite score was 53 (IQR: 39–67), from a range of 0–100, and EQ-5D-5L scores were 0.78 (IQR 0.65–0.90, utility value score) and 80 (70–88, VAS; Supplementary Table 9). Weak or no correlation was found between AGT-Eye subscale and each of NEI-VFQ-25 or EQ-5D-5L scores (Supplementary Table 10). Between the quality of life instruments, there was a weak to moderate correlation between NEI-VFQ-25 and each of EQ-5D-5L utility (ρ = 0.62 [95% CI: 0.56–0.68]) and visual analogue scale (ρ = 0.25 [95% CI: 0.16–0.34]) scores.

Regarding participants’ attitudes towards clinical trial participation, responses showed strong positive sentiments across various PACT-22 domains: positive beliefs (median score 88 [IQR: 75–100]), safety (75 [69–88]), information needs (88 [75–100]), and patient involvement (75 [62–88]; Supplementary Table 7). Negative expectations were low (46 [38–58]). Comparing AGT-Eye scores with participants’ attitudes towards clinical trial participation scored using PACT-22, a weak correlation was evident between knowledge of methods and each of positive beliefs (ρ = 0.30 [95% CI: 0.21–0.38]) and safety (ρ = 0.26 [95% CI: 0.18–0.35]), and between value of treatment and each of positive beliefs (ρ = 0.26 [95% CI: 0.17–0.34]), safety (ρ = 0.27 [95% CI: 0.19–0.36]), and information needs (ρ = 0.20 [95% CI: 0.11–0.28]). There was weak or no correlation between AGT-Eye subscale scores and the other PACT-22 domains (Supplementary Table 8).

### Comparison between current international survey and 2021 Australian survey data

#### Overall responses and instrument scores

Data from this multinational survey were compared to data from an Australian national survey of people with IRDs (*n* = 639) or their parents/caregivers (*n* = 42) [10, 11] administered in 2021. A similar number of individuals in both surveys had participated in medical research, but more participants from the USA had previously supplied DNA to a research database (75% compared to 60% in Australia, and 59% in other countries). Adults with IRDs in the USA had relatively higher NEI-VFQ-25 composite scores (mean (SD): 54.5 (19.8)) than those from Australia (49.6 (15.3)) and other countries (50.6 (18.7)). For PACT-22 domains, USA respondents had lower scores in the positive beliefs, safety, information needs, and patient involvement domains, but also less negative expectations than respondents from Australia and other countries (Supplementary Table 11). Over 90% of USA-based respondents are likely/very likely to get gene therapy if this was available to them, similar to 92% of respondents in Australia, and 93% in other global regions.

#### Comparison of AGT-Eye responses between USA and Australia

We compared AGT-Eye individual item responses between respondents from Australia and the USA, as USA residents constituted the major cohort from the international survey (Supplementary Table 12). Participants in the USA scored higher in the sources of information (mean (SD): 2.6 (0.7) versus 2.4 (0.9); adjusted *p* = 0.001) and knowledge of treatment methods (3.5 (0.5) versus 3.3 (0.4); *p* < 0.001) subscales, while Australian participants scored higher in the perceived value of treatment subscale (3.7 (0.5) versus 3.6 (0.6); *p* < 0.001).

Compared to Australian residents, respondents from the USA had higher self-rated knowledge about gene therapy for IRDs (41% vs 28% Australian reporting good knowledge; *p* = 0.007), and correctly indicated that gene therapy for the eye is not injected into the blood stream through the arm (45% vs 25% Australian respondents; *p* < 0.001) and that gene therapy and stem cell therapy are not the same treatment (68% vs 47% Australian residents; *p* < 0.001). For sources of information, USA residents reported research registries as a main information source (59% agreed vs 27% agreed in Australians, *p* < 0.001), and more Australian residents reported receiving information from family and friends (22% vs 17% in USA).

Regarding the perceived value of treatment, more Australian than US respondents believed that the government should pay all costs of gene therapy (43% versus 26%; *p* < 0.001), and that government subsidy of their treatment would be an effective use of taxpayer money (79% versus 64%; *p* < 0.001). There were no differences in participants’ willingness to travel to access gene therapy, or to consider a payment plan for gene therapy treatment.

## Discussion

This cross-sectional survey presents novel data on the international perspectives on gene therapy among people with IRDs and their parents/caregivers/carers. Responses were primarily from internet-enabled high-income English-speaking countries, and demonstrates geographic variation in participants’ knowledge, awareness, and perceived value of gene therapy treatment.

Using the AGT-Eye questionnaire [12], this study found that people with IRDs were generally willing to undergo gene therapy if this was available to them, despite knowledge gaps regarding the methods and potential outcomes of treatments. Another important finding is the uncertainty among IRD participants towards the impact of treatment on personal and government support. The international finding corroborates our previous study from IRD participants and parents/guardians in Australia [10, 11]. Key knowledge gaps were identified relating to treatment delivery method, with only 47% knowing that ocular gene therapy is not injected through the arm, and awareness of potential outcomes.

Compared to Australian data from 2021, more USA-based respondents self-reported they had good knowledge of gene therapy (41% vs 28%), which was reflected in their higher “knowledge of methods” subscale scores. Whereas voretigene neparvovec-rzyl (Luxturna®) was approved in Australia in 2021, the first IRD patient treated with ocular gene therapy was in the USA in 2007 [2, 18], which also has more clinical trials and research programs investigating regenerative IRD treatments [2]. USA-based respondents also received information from patient advocacy organisations, such as the Foundation Fighting Blindness, which has a patient-led registry (My Retina Tracker) launched in 2014 and has played a key role in driving research and patient education and facilitating access to emerging treatments [19].

From the international survey, weak to no correlation between AGT-Eye and each of NEI-VFQ-25 and EQ-5D-5L scores indicates that participants’ perception of ocular gene therapy, including their knowledge of the methods and outcomes, was not directly related to their health- and vision-related quality of life. These findings corroborate with that of our previous Australian National survey [10], suggesting that IRD patients have high interests towards ocular gene therapy regardless of their disease stage. PACT-22 responses showed strong positive sentiments towards clinical trial participation. The positive correlation between PACT-22 subdomain scores and AGT-Eye subdomain scores is not surprising, indicating that participants with higher knowledge of gene therapy and perceived value of treatment often also hold generally positive beliefs about clinical trials and assumptions that safeguards are in place. USA participants had relatively more guarded views regarding clinical trial involvement and safety, but also fewer negative expectations, than participants from other regions.

Most respondents reporting one or more perceived barriers to receiving gene therapy, the most common being cost (38%) and fear of side effects (27%). Despite these barriers and recognised knowledge gaps, 90% of the international respondents indicated that they were likely or very likely to take up gene therapy if this was available to them. Taken together with high positive beliefs and low negative expectation PACT-22 scores, present findings reflect high patient hopes for therapeutic interventions across IRD families globally.

Optimism towards emerging gene therapy treatments has been demonstrated in patients’ urgency to access therapies within a limited therapeutic window [20] and could be influenced by the dissemination of new treatments and discoveries through the general media [21]. A survey in China found that higher media use was associated with high acceptance of gene therapy among the public [22], and suggested that increasing access to media channels may positively affect potential recipient’s trust in innovative therapeutic approaches. However, mainstream media communication of ocular gene therapy can provide an overly optimistic view of timelines and often focus on curative language within a therapeutic spectrum [21]. Heightened patient expectations for therapeutic benefits could detracts from risks, cause disillusionment and despair among patient communities and, in the context of clinical trials, pose challenges to enrolment in ongoing and subsequent research. In patients treated with voretigene neparvovec-rzyl, sustained improvement in visual function has been reported in up to 7.5 years [23], but longer-term outcomes of ocular gene therapy are unknown. Despite most patients who participated in early phase *RPGR* gene therapy clinical trials not regretting their participation [24], increased patient education is vital to reduce potential therapeutic misestimation, so that families can make an informed and measured decision about new treatments.

From the present study, IRD participants favoured using the internet to access information. This corroborates with findings from our 2021 Australian IRD survey that internet was the commonest source where participants obtained information about gene therapy [10]. In contrast, previous research has shown that IRD patients would prefer to gain information about genetics and genetic testing from healthcare practitioners and websites of trusted agencies [25]. Less than 30% of our respondents reported receiving information about gene therapy from their ophthalmologists or other health care providers, highlighting the importance of clinician education and a need to change management paradigms and advice, in the light of emerging treatments. Multidisciplinary clinician education will help provide data to assist in these discussions, as well as enabling inter-profession communications and facilitating integrated care [26].

Beyond patients’ willingness to participate, publicity garnered by the FDA approval of voretigene neparvovec-rzyl has also led to high expectations for treatment efficacy in other IRDs [6], with potentially serious long-term consequences. Despite promising proof-of-concept studies and/or early phase results, a number of IRD gene therapies have either failed to progress to human trials or FDA approval, or have been terminated [2]. Contributing to these outcomes are unrealistic expectations of treatment efficacy, compounded by overly optimistic views of therapeutic benefit and lack of contextualisation of timeframes. Treatment outcomes also vary depending on the disease, and for most IRDs are likely to be less dramatic than the remarkable increases in retinal function observed in *RPE65*-related IRDs [27]. Ongoing efforts to promote open communication and pragmatic discussions between stakeholder groups (researchers, industry, patients, clinicians, and patient advocates) can provide a more nuanced understanding of potential visual outcomes from gene therapy treatments, and facilitate global efforts to advance the development of novel gene therapies.

Fewer USA than Australian respondents agreed that the government should pay all costs of their gene therapy and that it would be an effective use of taxpayer’s money (42% vs 26% and 79 vs 64%, respectively), which is likely reflective of differences in healthcare structures and policy, legislative, and reimbursement models. Australia’s universal healthcare model is a socialised system with responsibilities split between Federal and State governments [28]. In contrast, healthcare in the USA is non-centralized and provided through a combination of private health insurance and public health coverage [29]. In the case of voretigene neparvovec-rzyl, an outcome-based agreement between Harvard Pilgrim and Spark Therapeutics could improve treatment access and reduces the financial burden to patients [30], with rebates tied to both to both short and longer-term treatment outcomes. Nonetheless, present findings highlight disparities in perceived value of gene therapies, even between high-income countries with different health care structures. Barriers to adoption will be amplified in lower income countries and jurisdictions with existing disproportionate access to healthcare [22, 31].

Strengths of our study are its novelty as the first multinational survey, and recruitment came from a broad reaching global campaign through the assistance of patient support groups, resulting in a large number of responses. Limitations include using comparison Australian data collected in 2021, two years earlier and prior to the first Australian patient treated with voretigene neparvovec-rzyl. For the international survey, several AGT-Eye questions were updated to improve readability; thus, the comparison of subscale scores relating to knowledge is constrained by the choice of AGT-Eye items and wording. Knowledge levels were self-reported by participants and may not reflect their true knowledge status. Self-selected nature of participation, and mode of recruitment via patient support agencies, may have skewed results toward positive views of research. Although the survey was open to all countries, all our responses were from high- and middle-income countries. The survey was only administered in English, which may have limited access to people in other, non-English speaking regions. We included NEI-VFQ-25, EQ-5D-5L, and PACT-22 questionnaires to match our Australian National Survey protocol [11]; however, the additional time required to complete all instruments could have limited the overall response rates. In addition, the NEI-VFQ-25 measures the visual disability and symptoms on generic health and task-oriented domains and was used in several ocular gene therapy clinical trials [2]; however, the instrument was not designed to specifically capture vision-specific disability for IRDs [32]. Future evaluation with more recently developed IRD-specific patient-reported outcome measures may be more specific for informing vision-related disability in IRDs and associations with attitudes towards ocular gene therapy. Internet-only administration may also have limited access to some patient groups, which limits the generalizability of our findings to the broader population. Aside from the USA and UK, most countries had a small number of respondents. Thus, comparison data was limited to Australia and the USA.

## Conclusions

Findings from this study highlight the unique value of ocular gene therapy and its potential for transformative impact for families with IRDs. Knowledge gaps among potential gene therapy recipients include understanding clinical outcomes and financial implications. Between high income countries with different healthcare structures (USA and Australia), there were geographical variations in participants’ knowledge of gene therapy methods and perceived value of treatment, particularly pertaining to government reimbursement, but not in their willingness to travel to access gene therapy or to consider a payment plan for gene therapy treatment. Rising to the challenges of equitable access is a deeper issue than clinical translation. This is accentuated by the unique challenges of ocular gene therapy, as treatments often target small patient populations with severe unmet need, and there is often a delayed clinical benefit.

To promote shared decision making, considerations need to be given to both information needs and expectations of outcomes. Educational resources are needed for clinicians and potential candidates to facilitate discussions surrounding timelines and potential outcomes in relation to their patients’ vision loss and prognoses, risks, limitations of the effectiveness of the technology, and insurance/health reimbursement decisions against their potential therapeutic benefits. Patient and stakeholder perceptions of these therapies, alongside evidence of clinical and cost-effectiveness, will be vital to their eventual clinical adoption. Avenues for future research include examination of perspectives in lower income countries and the potential influences of religion, economic, and political differences between jurisdictions.

### Data access

All the study authors had full access to all the data in the study. ACBJ and LA takes responsibility for the integrity of the data and the accuracy of the data analysis.

### Supplementary information

Supplemental Figure and Supplemental Tables

## Data Availability

Non-identifiable data from this study are available from the corresponding author upon reasonable request.
