# Supplementary material for: A multinational survey of potential participant perspectives on ocular gene therapy
Source: Gene Ther. 2024 Apr 2;31(5-6):314–23. doi: 10.1038/s41434-024-00450-4 (PMC11090820; doi:10.1038/s41434-024-00450-4)
Supplement: Supplementary file 1 — Supplemental Figure and Supplemental Tables [file 41434_2024_450_MOESM1_ESM.pdf]

<u>Supplementary Materials:</u>

A multinational survey of potential participant perspectives on ocular gene therapy.

Britten-Jones AC, McGuinness MB, Chen FK, Grigg JR, Mack HG, Ayton LN.

Supplement Figure 1: Participant flow

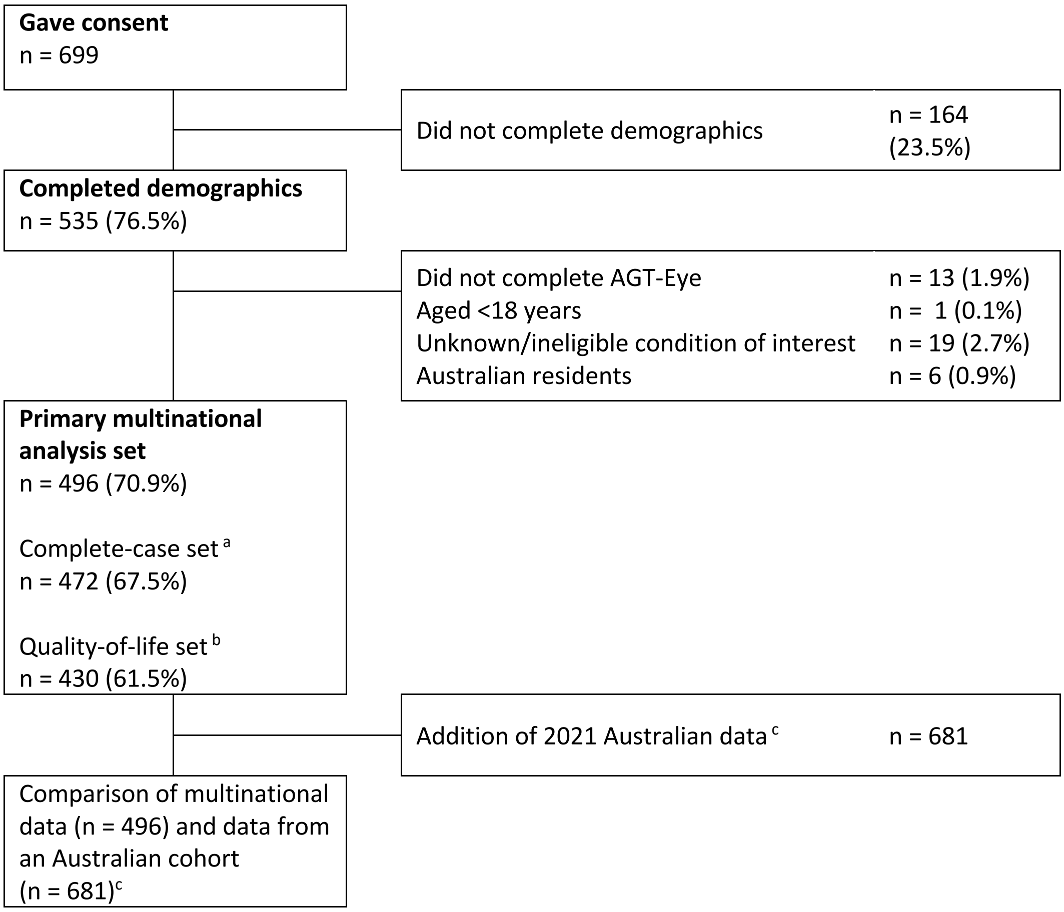

[a] Participants with non-missing data for all instruments. The complete-case set was used for the comparison of AGT-eye and PACT-22 subscale scores.
[b] Adults with IRDs who completed both the NEI-VFQ-25 and EQ-5D-5L. The quality-of-life set was used for the comparison of AGT-eye scores and quality-of-life instrument scores.
[c] Data from an Australian cohort as published in Mack et al., 2023.[10]

Supplemental Table 1. Participants' self-reported inherited retinal diseases diagnoses

**Widespread IRDs**	**Predominantly central IRDs**
ABCA4	Bull's eye maculopathy
Fundus flavimaculatus	Leber congenital amaurosis
Abetalipoproteinaemia	Macular dystrophy
Achromatopsia	Pattern dystrophy
Rod monochromatism	Sorsby fundus dystrophy
Bietti Crystalline Dystrophy	Stargardt disease
Bull's eye retinal dystrophy	Vitelliform macular dystrophy
Central areolar choroidal dystrophy	Adult vitelliform macular degeneration
CEP47	Best disease
Choroideremia	X-Linked retinoschisis
Cone-rod dystrophy	Malattia Leventinese
X-linked cone rod dystrophy	
Cone dystrophy	
Congenital stationary night blindness	
Doyne honeycomb retinal dystrophy	
Familial exudative vitreoretinopathy	
Goldmann-Favre syndrome	
Gyrate atrophy	
PRPH2-associated retinopathy	
Retinal dystrophy	
Retinitis pigmentosa	
Laurence-Moon-Bardet-Biedl Syndrome	
Usher syndrome	
Vitreoretinochoroidopathy	

Excluded conditions: Age-related macular degeneration; Branch retinal artery occlusion; Cystoid macular oedema; Glaucoma; Myopic degeneration; Pseudoxanthoma elasticum; Thinning retina; Unknown retinal diseases.

Supplemental Table 2. Differences between Australian version and the multinational version of the AGT-Eye instrument

Item	Australian version	Multinational version
3	I understand the difference between an experimental treatment provided in a clinical trial and a treatment that has already been approved by the Australian Government.	I understand the difference between an experimental treatment provided in a clinical trial and a treatment that has already been approved by the government.
4	Gene therapy for the eye is suitable at any stage of a person's life.	Gene therapy for the eye is suitable for any stage of disease progression and vision loss.
8	Gene therapy for the eye can restore vision back to normal.	The main goal of ocular gene therapy is to restore vision back to normal.
9	Gene therapy for the eye is a treatment that may slow down the disease.	The main goal of ocular gene therapy is to slow down the disease.
17	If I undergo gene therapy, it will affect my eligibility or terms of conditions in life, disability or health insurance in the future.	If I undergo gene therapy, it will affect my eligibility or terms of conditions in life, disability or health/medical insurance in the future.
20	If gene therapy for my condition was not available in my state I would consider travelling interstate to access it.	If gene therapy for my condition was not available in my local area, I would consider travelling to another country to access it.
21	My private health insurance should pay all out of pocket costs for my gene therapy.	My private health/medical insurance should pay all out of pocket costs for my gene therapy.

Supplemental Table 3. Additional demographics characteristics of the multinational survey cohort

	Participant type		
	**Adult with IRD** 439 (88.5%)	**Parent/guardian/carer** 57 (11.5%)	**Total** 496 (100.0%)
**World Bank country income status**			
High	422 (96.1%)	53 (93.0%)	475 (95.8%)
Upper middle	15 (3.4%)	2 (3.5%)	17 (3.4%)
Lower middle	2 (0.5%)	2 (3.5%)	4 (0.8%)
**Primary language**			
English	393 (89.5%)	37 (64.9%)	430 (86.7%)
Arabic	2 (0.5%)	2 (3.5%)	4 (0.8%)
Chinese (Mandarin, Cantonese, or local dialect)	2 (0.5%)	0 (0.0%)	2 (0.4%)
French	2 (0.5%)	1 (1.8%)	3 (0.6%)
Hindi	1 (0.2%)	0 (0.0%)	1 (0.2%)
Portuguese	1 (0.2%)	0 (0.0%)	1 (0.2%)
Spanish	12 (2.7%)	6 (10.5%)	18 (3.6%)
Italian	6 (1.4%)	2 (3.5%)	8 (1.6%)
German	6 (1.4%)	0 (0.0%)	6 (1.2%)
Dutch	5 (1.1%)	0 (0.0%)	5 (1.0%)
Polish	2 (0.5%)	4 (7.0%)	6 (1.2%)
Hungarian	2 (0.5%)	0 (0.0%)	2 (0.4%)
Greek	0 (0.0%)	1 (1.8%)	1 (0.2%)
Swedish	2 (0.5%)	0 (0.0%)	2 (0.4%)
Norwegian	1 (0.2%)	0 (0.0%)	1 (0.2%)
Tamil	0 (0.0%)	1 (1.8%)	1 (0.2%)
Afrikaans	1 (0.2%)	2 (3.5%)	3 (0.6%)
Romanian	1 (0.2%)	0 (0.0%)	1 (0.2%)
ASL	0 (0.0%)	1 (1.8%)	1 (0.2%)
**Time period of most recent decline in vision**			
No decline, stable vision	44 (10%)	20 (35%)	64 (13%)
Less than 6 months	54 (12%)	9 (16%)	63 (13%)
1 Year	100 (23%)	8 (14%)	108 (22%)
5 Years	147 (33%)	14 (25%)	161 (32%)
10 Years	94 (21%)	6 (11%)	100 (20%)
**Initial symptoms selected**			
Difficulty seeing at night or dusk	303 (69%)	24 (42%)	327 (66%)
Bumping into low lying objects	137 (31%)	12 (21%)	149 (30%)
Difficulty driving	131 (30%)	2 (4%)	133 (27%)
Difficulty adjusting from light to dark and vice versa	225 (51%)	18 (32%)	243 (49%)
Missing parts in vision	148 (34%)	15 (26%)	163 (33%)
Noticed peripheral or side vision reducing	157 (36%)	14 (25%)	171 (34%)
Other	41 (9%)	12 (21%)	53 (11%)
No noticeable symptoms	16 (4%)	8 (14%)	24 (5%)
Can't recall	10 (2%)	1 (2%)	11 (2%)
**Perceived barriers to gene therapy for IRDs[a]**			
Early phase of roll out	95 (22%)	15 (26%)	110 (22%)
May not work	75 (17%)	15 (26%)	90 (18%)
Fear of side effects	117 (27%)	17 (30%)	134 (27%)
Cost	170 (39%)	17 (30%)	187 (38%)
Religion/personal belief	3 (1%)	2 (4%)	5 (1%)
Loss of government support	11 (3%)	1 (2%)	12 (2%)
None	144 (33%)	16 (28%)	160 (32%)

Data presented as frequency (%) of responses. [a]Multiple symptoms and barriers could be selected

Supplemental Table 4. Frequency of responses to AGT-Eye items (n=496)

	Item	Strongly disagree	Disagree	Neither agree/disagree	Agree	Strongly agree
	**Sources of information**					
2a	My ophthalmologist	105 (20.9)	166 (33.1)	77 (15.3)	111 (22.1)	37 (7.4)
2b	Other medical or health professional	104 (20.7)	169 (33.7)	81 (16.1)	99 (19.7)	43 (8.6)
2c	Registry (e.g., Foundation Fighting Blindness)	44 (8.8)	63 (12.5)	90 (17.9)	171 (34.1)	128 (25.5)
2d	Research group	82 (16.3)	156 (31.1)	108 (21.5)	104 (20.7)	46 (9.2)
2e	Newspapers	132 (26.3)	173 (34.5)	91 (18.1)	86 (17.1)	14 (2.8)
2f	Internet	39 (7.8)	87 (17.3)	64 (12.7)	222 (44.2)	84 (16.7)
2g	Social media	152 (30.3)	172 (34.3)	68 (13.5)	86 (17.1)	18 (3.6)
2h	Patient support group	135 (26.9)	191 (38.0)	75 (14.9)	70 (13.9)	25 (5.0)
2i	Family/friends	138 (27.5)	196 (39.0)	75 (14.9)	71 (14.1)	16 (3.2)
	**Knowledge of methods**					
1	I have good knowledge about gene therapy for inherited retinal diseases	36 (7.2)	90 (17.9)	143 (28.5)	160 (31.9)	67 (13.3)
3	I understand the difference between an experimental treatment provided in a clinical trial and a treatment that has already been approved by the government	5 (1.0)	14 (2.8)	18 (3.6)	179 (35.7)	280 (55.8)
4	Gene therapy for the eye is suitable for any stage of disease progression and vision loss	24 (4.8)	118 (23.5)	220 (43.8)	97 (19.3)	37 (7.4)
5	Generally, gene therapy for inherited retinal disease is delivered to both eyes	17 (3.4)	87 (17.3)	241 (48.0)	115 (22.9)	36 (7.2)
6	Gene therapy for the eye is injected into the blood stream through the arm	95 (18.9)	141 (28.1)	232 (46.2)	22 (4.4)	6 (1.2)
7	Gene therapy and stem cell therapy are the same treatment	128 (25.5)	223 (44.4)	131 (26.1)	11 (2.2)	3 (0.6)
	**Awareness of outcomes**					
8	The main goal of ocular gene therapy is to restore vision back to normal	34 (6.8)	181 (36.1)	177 (35.3)	88 (17.5)	16 (3.2)
9	The main goal of ocular gene therapy is to slow down the disease	1 (0.2)	31 (6.2)	150 (29.9)	265 (52.8)	49 (9.8)
10	Treatment complications to my eyes, such as permanent blindness, are possible with an approved gene therapy	12 (2.4)	53 (10.6)	181 (36.1)	205 (40.8)	45 (9.0)
11	Gene therapy in my eye may have side effects elsewhere in my body	9 (1.8)	78 (15.5)	237 (47.2)	151 (30.1)	21 (4.2)
12	Having gene therapy for their eye condition means a person will not pass on an eye condition to any children they may have in the future	185 (36.9)	201 (40.0)	88 (17.5)	16 (3.2)	6 (1.2)

13	I may not be eligible for financial or other government benefits if my gene therapy for my eye condition is successful	26 (5.2)	64 (12.7)	241 (48.0)	128 (25.5)	37 (7.4)
14	Gene therapy for inherited retinal diseases will require many years of follow-up with my eyecare practitioner	2 (0.4)	21 (4.2)	120 (23.9)	244 (48.6)	109 (21.7)
15	Receiving gene therapy for my inherited retinal disease means I won't be eligible for future genetic treatments	8 (1.6)	145 (28.9)	263 (52.4)	68 (13.5)	12 (2.4)
16	I will lose my privacy if I undergo gene therapy, and my data will be in the public domain	111 (22.1)	218 (43.4)	137 (27.3)	26 (5.2)	4 (0.8)
17	If I undergo gene therapy, it will affect my eligibility or terms of conditions in life, disability or health/medical insurance in the future	39 (7.8)	147 (29.3)	251 (50.0)	48 (9.6)	11 (2.2)
	**Perceived value of treatment**					
18	The government should pay all costs of my gene therapy	20 (4.0)	96 (19.1)	201 (40.0)	107 (21.3)	72 (14.3)
19	Government subsidy of my treatment would be an effective use of taxpayer money	12 (2.4)	28 (5.6)	124 (24.7)	212 (42.2)	120 (23.9)
20	If gene therapy for my condition was not available in my local area, I would consider travelling to another country to access it	7 (1.4)	58 (11.6)	59 (11.8)	200 (39.8)	172 (34.3)
21	My private health/medical insurance should pay all out of pocket costs for my gene therapy	18 (3.6)	61 (12.2)	164 (32.7)	165 (32.9)	88 (17.5)
22	I would consider a payment plan for my gene therapy	19 (3.8)	41 (8.2)	116 (23.1)	234 (46.6)	86 (17.1)

No AGT-eye items were reverse coded prior to tabulation.

Supplemental Table 5. Multinational AGT-Eye subscale scores according to respondent characteristics

	n	Information sources		Knowledge of methods		Awareness of outcomes		Perceived value of treatment	
		Mean (SD)	p-value	Mean (SD)	p-value	Mean (SD)	p-value	Mean (SD)	p-value
**Age**			0.205		0.366		**<0.0001**		**0.002**
18 to 39 years	81	2.8 (0.7)		3.6 (0.5)		**3.5 (0.3)**		**3.7 (0.6)**	
40 to 59 years	217	2.7 (0.7)		3.6 (0.5)		**3.4 (0.3)**		**3.7 (0.6)**	
60+ years	198	2.6 (0.8)		3.5 (0.5)		**3.3 (0.3)**		**3.5 (0.6)**	
**Participant type**			**0.003**		**0.036**		0.213		0.307
Adult with IRD	439	**2.7 (0.7)**		**3.5 (0.5)**		3.4 (0.3)		3.6 (0.6)	
Parent/guardian/carer	57	**3.0 (0.6)**		**3.7 (0.5)**		3.4 (0.3)		3.7 (0.6)	
**Gender**			0.544		0.786		0.863		0.207
Male	237	2.7 (0.7)		3.6 (0.5)		3.4 (0.3)		3.7 (0.5)	
Female	255	2.7 (0.8)		3.5 (0.4)		3.4 (0.3)		3.6 (0.6)	
**Retinal region involved**			0.288		0.783		0.110		0.925
Widespread retinal involvement	366	2.7 (0.7)		3.6 (0.5)		3.4 (0.3)		3.6 (0.6)	
Predominantly central	130	2.6 (0.8)		3.6 (0.5)		3.4 (0.3)		3.6 (0.5)	
**Likelihood of taking up gene therapy**			**0.037**		0.195		**0.025**		**<0.001**
Unlikely/Very unlikely	6	**2.6 (0.5)**		3.5 (0.4)		**3.1 (0.4)**		**2.6 (0.9)**	
Neutral	39	**2.4 (0.7)**		3.4 (0.5)		**3.5 (0.3)**		**3.4 (0.5)**	
Likely/Very likely	451	**2.7 (0.7)**		3.6 (0.5)		**3.4 (0.3)**		**3.7 (0.6)**	
**Highest level of education** [a]			0.878		**0.013**		0.095		**0.014**
Primary school (up to 12 years of age)	18	2.6 (0.7)		**3.3 (0.5)**		3.3 (0.3)		**3.3 (0.6)**	
Secondary school (until at least 15 years of age)	60	2.7 (0.8)		**3.5 (0.5)**		3.4 (0.3)		**3.6 (0.6)**	
Trade certificate	71	2.8 (0.7)		**3.5 (0.5)**		3.4 (0.4)		**3.7 (0.5)**	
Bachelor degree	168	2.7 (0.7)		**3.6 (0.5)**		3.4 (0.3)		**3.6 (0.6)**	
Post-graduate degree	159	2.7 (0.8)		**3.6 (0.5)**		3.5 (0.3)		**3.7 (0.6)**	
I prefer not to say [a]	20	2.8 (0.6)		**3.4 (0.5)**		3.3 (0.2)		**3.4 (0.6)**	
**Self-reported eyesight**			0.768		0.268		0.903		0.354
Excellent	9	2.8 (0.7)		3.8 (0.5)		3.4 (0.3)		3.5 (0.6)	
Good	87	2.8 (0.7)		3.6 (0.5)		3.4 (0.3)		3.6 (0.6)	
Fair	146	2.6 (0.8)		3.5 (0.5)		3.4 (0.4)		3.6 (0.6)	
Poor	132	2.7 (0.7)		3.6 (0.5)		3.4 (0.3)		3.6 (0.6)	
Very poor	98	2.7 (0.7)		3.6 (0.5)		3.4 (0.4)		3.7 (0.6)	
Completely blind	19	2.9 (0.7)		3.7 (0.5)		3.4 (0.4)		3.8 (0.5)	

Scores not shown for categories with <5 observations. p-values from one-way ANOVA. Statistically significant findings are shown in **bold**. [a] p-values calculations excluded responses that were "I prefer not to say".

Supplemental Table 6. Correlation between AGT-Eye subscale scores in the multinational cohort(n=496)

	**AGT-Eye subscale**					
	**Information sources**		**Knowledge of methods**		**Awareness of outcomes**	
	ρ	(95% CI)	ρ	(95% CI)	ρ	(95% CI)
Information sources	1.00					
Knowledge of Methods	**0.50**	**(0.43,0.56)**	1.00			
Awareness of outcomes	**0.14**	**(0.05,0.23)**	**0.29**	**(0.20,0.37)**	1.00	
Perceived value of treatment	**0.16**	**(0.07,0.24)**	**0.19**	**(0.10,0.27)**	**0.12**	**(0.03,0.20)**

ρ = Spearman's correlation coefficient. Statistically significant correlations are shown in **bold**.

Supplemental Table 7. Distribution of PACT-22 domain scores

	Participant type		
	**Adult with IRD**	**Parent/guardian/carer**	**Total**
	n=422 (89%)	n=50 (11%)	n=472 (100%)
Positive beliefs	88 [75, 100]	88 [75, 100]	88 [75, 100]
Safety	75 [69, 88]	75 [69, 88]	75 [69, 88]
Information needs	88 [75, 100]	88 [75, 100]	88 [75, 100]
Negative expectations	50 [38, 58]	38 [29, 46]	46 [38, 58]
Patient involvement	75 [62, 88]	75 [62, 94]	75 [62, 88]

Values are Median [IQR].

Supplemental Table 8. Correlation between AGT-Eye subscale scores and PACT-22 domains (n=477)

	**Information sources**		**Knowledge of methods**		**Awareness of outcomes**		**Value of treatment**	
Positive beliefs	0.08	(-0.01,0.17)	**0.30**	**(0.21,0.38)**	**0.15**	**(0.06,0.24)**	**0.26**	**(0.17,0.34)**
Safety	**0.18**	**(0.10,0.27)**	**0.26**	**(0.18,0.35)**	**0.19**	**(0.10,0.27)**	**0.27**	**(0.19,0.36)**
Information needs	0.02	(-0.07,0.11)	**0.12**	**(0.03,0.21)**	0.06	(-0.04,0.14)	**0.20**	**(0.11,0.28)**
Negative expectations	0.00	(-0.09,0.09)	**0.17**	**(0.08,0.26)**	**0.14**	**(0.05,0.23)**	**-0.12**	**(-0.21,-0.03)**
Patient involvement	**0.12**	**(0.03,0.20)**	**0.11**	**(0.02,0.20)**	-0.02	(-0.11,0.07)	**0.28**	**(0.20,0.37)**

Rho = Spearman's correlation coefficient. Statistically significant correlations are shown in **bold**.

Supplemental Table 9. Distribution of quality-of-life instrument scores

	Participant type		
	**Adult with IRD** n=430 (89%)	**Parent/guardian/carer** n=53 (11%)	**Total** n=483 (100%)
**NEI-VFQ-25**			
Composite score	53 [39, 66]	59 [40, 81]	53 [39, 67]
General health	75 [50, 75]	75 [75, 100]	75 [50, 75]
General vision	60 [40, 60]	40 [20, 80]	40 [40, 60]
Ocular pain	88 [75, 100]	88 [75, 100]	88 [75, 100]
Near activities	50 [33, 75]	50 [25, 83]	50 [33, 75]
Distance activities	50 [33, 67]	50 [25, 75]	50 [33, 67]
Vision-specific social functioning	62 [38, 75]	75 [38, 100]	62 [38, 75]
Vision-specific mental health	44 [25, 62]	56 [25, 81]	44 [25, 69]
Vision-specific role difficulties	50 [25, 75]	50 [38, 75]	50 [25, 75]
Vision-specific dependency	58 [33, 75]	67 [42, 83]	58 [33, 75]
Driving	0 [0, 50]	0 [0, 67]	0 [0, 50]
Colour vision	75 [50, 100]	75 [50, 100]	75 [50, 100]
Peripheral vision	25 [25, 75]	50 [25, 75]	25 [25, 75]
**EQ-5D-5L utility value from US value set**	0.78 [0.65, 0.88]	0.84 [0.69, 0.93]	0.78 [0.65, 0.90]
**EQ Visual Analogue Scale score**	80 [70, 85]	90 [80, 93]	80 [70, 88]

Values are Median [IQR]. Missing quality-of-life scores for 8 participants who were included in the primary analysis set.

Supplemental Table 10. Correlation between AGT-Eye subscales and quality-of-life instruments among adult participants with an inherited retinal disease (n=434)

	**NEI-VFQ-25 composite score**		**EQ-5D-5L utility score**		**EQ-VAS**	
Sources of information	-0.07	(-0.17,0.02)	-0.08	(-0.17,0.01)	0.02	(-0.07,0.12)
Knowledge of methods	-0.08	(-0.17,0.02)	**-0.11**	**(-0.20,-0.02)**	0.07	(-0.03,0.16)
Awareness of outcomes	-0.04	(-0.14,0.05)	**-0.10**	**(-0.20,-0.01)**	-0.08	(-0.18,0.01)
Perceived value of treatment	**-0.18**	**(-0.27,-0.09)**	**-0.13**	**(-0.22,-0.04)**	0.00	(-0.09,0.10)
NEI-VFQ-25 composite score	1.00					
EQ-5D-5L utility	**0.62**	**(0.56,0.68)**	1.00			
EQ Visual Analogue Scale	**0.26**	**(0.17,0.34)**	**0.43**	**(0.35,0.51)**	1.00	

Rho = Spearman's correlation coefficient. Statistically significant correlations are shown in **bold**.

Supplemental Table 11. Combined participant responses from the multinational survey (n=496) and data from an Australian National survey of people with IRDs (n=681)

**Characteristic**	**Participant type**					
	**Adult**			**Parent/Guardian/Carer**		
	**Australia** n=639	**USA** n=316	**Other** n=123	**Australia** n=42	**USA** n=32	**Other** n=25
**Age,** years [a]	54.1 (15.9)	57.4 (14.2)	51.2 (14.1)	44.5 (10.7)	46.7 (11.0)	43.5 (11.0)
**Previously supplied DNA to a research IRD database**	381 (60%)	234 (74%)	76 (62%)	31 (74%)	27 (84%)	12 (48%)
**Previously participated in medical research**	186 (29%)	86 (27%)	34 (28%)	7 (17%)	11 (34%)	2 (8.0%)
**Likelihood of taking up gene therapy**						
Likely/Very likely	586 (92%)	284 (90%)	115 (93%)	38 (90%)	30 (94%)	22 (88%)
Neutral	40 (6.3%)	28 (8.9%)	7 (5.7%)	3 (7.1%)	2 (6.3%)	2 (8.0%)
Unlikely/Very unlikely	13 (2.0%)	4 (1.3%)	1 (0.8%)	1 (2.4%)	0 (0%)	1 (4.0%)
**AGT-Eye scores** [a]						
Sources of information	2.4 (0.9)	2.6 (0.7)	2.8 (0.8)	2.6 (0.8)	2.9 (0.6)	3.0 (0.5)
Knowledge of methods	3.3 (0.4)	3.5 (0.5)	3.7 (0.5)	3.4 (0.5)	3.7 (0.6)	3.7 (0.3)
Awareness of outcomes	3.4 (0.3)	3.4 (0.3)	3.4 (0.4)	3.4 (0.2)	3.4 (0.3)	3.3 (0.3)
Perceived value of treatment	3.7 (0.5)	3.6 (0.6)	3.8 (0.6)	4.0 (0.4)	3.7 (0.6)	3.7 (0.7)
**PACT22 scores** [a]						
Positive beliefs	88.7 (12.0)	86.0 (13.3)	89.6 (12.6)	90.3 (11.1)	86.3 (12.3)	83.0 (12.7)
Safety	83.5 (13.8)	74.8 (14.8)	83.1 (14.8)	85.7 (14.8)	78.3 (13.7)	74.7 (13.5)
Information needs	85.6 (13.6)	84.2 (13.4)	87.7 (12.7)	89.9 (11.8)	86.5 (12.0)	83.0 (13.1)
Negative expectations	44.5 (19.0)	51.2 (17.1)	42.5 (18.1)	38.1 (15.3)	40.3 (16.1)	36.0 (10.4)
Patient involvement	78.0 (15.3)	76.1 (14.3)	78.4 (15.5)	77.8 (14.6)	76.7 (16.2)	75.9 (13.8)
**Quality of life instrument scores** [a]						
**NEI-VFQ-25 composite score**	49.6 (15.3)	54.5 (19.8)	50.6 (18.7)	52.5 (17.7)	56.9 (24.4)	60.2 (24.8)
**EQ-5D-5L utility value** [b]	0.8 (0.2)	0.8 (0.2)	0.7 (0.2)	0.8 (0.2)	0.8 (0.2)	0.8 (0.2)
**EQ Visual Analogue Scale**	73.1 (18.1)	77.5 (13.6)	71.4 (18.9)	82.0 (16.2)	81.5 (16.6)	87.4 (11.7)

[a] Data presented as Mean (SD) or frequency of responses (%) as indicated.
[b] EQ-5D-5L utility values were calculated from the Australian value set for Australian data and from the US value set for all other regions.

Supplemental Table 12: Comparison of AGT-Eye subscale scores and item responses from between IRD respondents from the USA and Australia.

	Item	Australia n=681	USA n=348	Unadjusted p-value[a]
	**Sources of information**			
	**Subscale score**	2.4 (0.9)	2.6 (0.7)	0.001
2a	**My ophthalmologist**			0.003
	Strongly agree/Agree	258 (38%)	102 (29%)	
	Neither agree or disagree	122 (18%)	53 (15%)	
	Strongly disagree/Disagree	301 (44%)	**193 (55%)**	
2b	**Other medical or health professional**			0.008
	Strongly agree/Agree	131 (19%)	96 (28%)	
	Neither agree or disagree	125 (18%)	53 (15%)	
	Strongly disagree/Disagree	425 (62%)	**199 (57%)**	
2c	**Registry**			<0.001
	Strongly agree/Agree	187 (27%)	**206 (59%)**	
	Neither agree or disagree	136 (20%)	65 (19%)	
	Strongly disagree/Disagree	**358 (53%)**	77 (22%)	
2d	**Research group**			0.2
	Strongly agree/Agree	162 (24%)	93 (27%)	
	Neither agree or disagree	145 (21%)	83 (24%)	
	Strongly disagree/Disagree	**374 (55%)**	172 (49%)	
2e	**Newspapers**			0.5
	Strongly agree/Agree	109 (16%)	59 (17%)	
	Neither agree or disagree	142 (21%)	62 (18%)	
	Strongly disagree/Disagree	**430 (63%)**	**227 (65%)**	
2f	**Internet**			0.03
	Strongly agree/Agree	336 (49%)	**202 (58%)**	
	Neither agree or disagree	110 (16%)	47 (14%)	
	Strongly disagree/Disagree	235 (35%)	99 (28%)	
2g	**Social media**			0.039
	Strongly agree/Agree	114 (17%)	62 (18%)	
	Neither agree or disagree	122 (18%)	41 (12%)	
	Strongly disagree/Disagree	**445 (65%)**	**245 (70%)**	
2h	**Patient support group**			0.2
	Strongly agree/Agree	95 (14%)	48 (14%)	
	Neither agree or disagree	134 (20%)	53 (15%)	
	Strongly disagree/Disagree	**452 (66%)**	**247 (71%)**	
2i	**Family/friends**			<0.001
	Strongly agree/Agree	149 (22%)	60 (17%)	
	Neither agree or disagree	154 (23%)	51 (15%)	
	Strongly disagree/Disagree	**378 (56%)**	**237 (68%)**	
	**Knowledge of methods**			
	**Subscale score**	3.3 (0.4)	3.5 (0.5)	<0.001
1	**I have good knowledge about gene therapy for inherited retinal diseases**			<0.001
	Strongly agree/Agree	193 (28%)	141 (41%)	
	Neither agree or disagree	235 (35%)	111 (32%)	
	Strongly disagree/Disagree	253 (37%)	96 (28%)	
3	**I understand the difference between an experimental treatment provided in a clinical trial and a treatment that has already been approved by the government**[d]			0.014
	Strongly agree/Agree	**592 (87%)**	**319 (92%)**	
	Neither agree or disagree	59 (8.7%)	13 (3.7%)	

	Strongly disagree/Disagree	30 (4.4%)	16 (4.6%)	
4	***Gene therapy for the eye is suitable for any stage of disease progression and vision loss*** [c]			<0.001
	Strongly agree/Agree	**349 (51%)**	91 (26%)	
	Neither agree or disagree	272 (40%)	162 (47%)	
	Strongly disagree/Disagree	60 (8.8%)	95 (27%)	
5	**Generally, gene therapy for inherited retinal disease is delivered to both eyes**			<0.001
	Strongly agree/Agree	289 (42%)	92 (26%)	
	Neither agree or disagree	**338 (50%)**	**180 (52%)**	
	Strongly disagree/Disagree	54 (7.9%)	76 (22%)	
6	***Gene therapy for the eye is injected into the blood stream through the arm***			<0.001
	Strongly agree/Agree	89 (13%)	16 (4.6%)	
	Neither agree or disagree	**421 (62%)**	**177 (51%)**	
	Strongly disagree/Disagree	171 (25%)	155 (45%)	
7	***Gene therapy and stem cell therapy are the same treatment***			<0.001
	Strongly agree/Agree	52 (7.6%)	8 (2.3%)	
	Neither agree or disagree	308 (45%)	103 (30%)	
	Strongly disagree/Disagree	321 (47%)	**237 (68%)**	
	**Awareness of outcomes**			
	**Subscale score**	3.4 (0.3)	3.4 (0.3)	0.14
8	***The main goal of ocular gene therapy is to restore vision back to normal*** [d]			<0.001
	Strongly agree/Agree	76 (11%)	69 (20%)	
	Neither agree or disagree	**348 (51%)**	129 (37%)	
	Strongly disagree/Disagree	257 (38%)	150 (43%)	
9	**The main goal of ocular gene therapy is to slow down the disease** [e]			<0.001
	Strongly agree/Agree	**472 (69%)**	**214 (61%)**	
	Neither agree or disagree	202 (30%)	115 (33%)	
	Strongly disagree/Disagree	7 (1.0%)	19 (5.5%)	
10	**Treatment complications to my eyes, such as permanent blindness, are possible with an approved gene therapy**			<0.001
	Strongly agree/Agree	216 (32%)	175 (50%)	
	Neither agree or disagree	**388 (57%)**	134 (39%)	
	Strongly disagree/Disagree	77 (11%)	39 (11%)	
11	**Gene therapy in my eye may have side effects elsewhere in my body**			<0.001
	Strongly agree/Agree	162 (24%)	126 (36%)	
	Neither agree or disagree	**416 (61%)**	164 (47%)	
	Strongly disagree/Disagree	103 (15%)	58 (17%)	
12	**Having gene therapy for their eye condition means a person will not pass on an eye condition to any children they may have in the future**			<0.001
	Strongly agree/Agree	39 (5.7%)	14 (4.0%)	
	Neither agree or disagree	220 (32%)	56 (16%)	
	Strongly disagree/Disagree	**422 (62%)**	**278 (80%)**	
13	**I may not be eligible for financial or other government benefits if my gene therapy for my eye condition is successful**			<0.001
	Strongly agree/Agree	267 (39%)	99 (28%)	
	Neither agree or disagree	**345 (51%)**	**185 (53%)**	
	Strongly disagree/Disagree	69 (10%)	64 (18%)	

14	**Gene therapy for inherited retinal diseases will require many years of follow-up with my eyecare practitioner**			0.2
	Strongly agree/Agree	**456 (67%)**	**251 (72%)**	
	Neither agree or disagree	207 (30%)	87 (25%)	
	Strongly disagree/Disagree	18 (2.6%)	10 (2.9%)	
15	**Receiving gene therapy for my inherited retinal disease means I won't be eligible for future genetic treatments**			<0.001
	Strongly agree/Agree	37 (5.4%)	46 (13%)	
	Neither agree or disagree	**431 (63%)**	**199 (57%)**	
	Strongly disagree/Disagree	213 (31%)	103 (30%)	
16	**I will lose my privacy if I undergo gene therapy, and my data will be in the public domain**			0.051
	Strongly agree/Agree	50 (7.3%)	20 (5.7%)	
	Neither agree or disagree	152 (22%)	101 (29%)	
	Strongly disagree/Disagree	**479 (70%)**	**227 (65%)**	
17	**If I undergo gene therapy, it will affect my eligibility or terms of conditions in life, disability or health/medical insurance in the future**			0.3
	Strongly agree/Agree	87 (13%)	34 (9.8%)	
	Neither agree or disagree	**368 (54%)**	**187 (54%)**	
	Strongly disagree/Disagree	226 (33%)	127 (36%)	
	**Perceived value of treatment**			
	**Subscale score**	3.7 (0.5)	3.6 (0.6)	<0.001
18	**The government should pay all costs of my gene therapy**			<0.001
	Strongly agree/Agree	292 (43%)	89 (26%)	
	Neither agree or disagree	275 (40%)	161 (46%)	
	Strongly disagree/Disagree	114 (17%)	98 (28%)	
19	**Government subsidy of my treatment would be an effective use of taxpayer money**			<0.001
	Strongly agree/Agree	**538 (79%)**	**221 (64%)**	
	Neither agree or disagree	126 (19%)	93 (27%)	
	Strongly disagree/Disagree	17 (2.5%)	34 (9.8%)	
20	**If gene therapy for my condition was not available in my local area, I would consider travelling to another country to access it** [f]			0.015
	Strongly agree/Agree	**521 (77%)**	**249 (72%)**	
	Neither agree or disagree	102 (15%)	49 (14%)	
	Strongly disagree/Disagree	58 (8.5%)	50 (14%)	
21	**My private health/medical insurance should pay all out of pocket costs for my gene therapy**			0.003
	Strongly agree/Agree	314 (46%)	**191 (55%)**	
	Neither agree or disagree	284 (42%)	107 (31%)	
	Strongly disagree/Disagree	83 (12%)	50 (14%)	
22	**I would consider a payment plan for my gene therapy**			0.2
	Strongly agree/Agree	**424 (62%)**	**226 (65%)**	
	Neither agree or disagree	196 (29%)	83 (24%)	
	Strongly disagree/Disagree	61 (9.0%)	39 (11%)	

Values are frequency of responses (%) and mean (SD) for subscale scores. Frequencies over 50% are shown in bold. No items were reverse coded prior to tabulation. AGT-Eye items that were reverse coded for scoring (i.e., higher scores given to disagree) are italicised.

[a] p-values are calculated using the Pearson's Chi-squared test or two-sample t-test.

[b] Adjusted alpha = 0.0017 under Bonferroni correction for 30 tests and a family wise type I error probability of 5%.

[c] Differences in Australian version wording: Gene therapy for the eye is suitable at any stage of a person's life.

[d] Differences in Australian version wording: Gene therapy for the eye can restore vision back to normal.

[e] Differences in Australian version wording: Gene therapy for the eye is a treatment that may slow down the disease.

[f] Differences in Australian version wording: If gene therapy for my condition was not available in my state I would consider travelling interstate to access it.
